# Intraoperative milrinone versus dobutamine in cardiac surgery patients: a retrospective cohort study on mortality

**DOI:** 10.1186/s13054-018-1969-1

**Published:** 2018-02-26

**Authors:** Dorthe Viemose Nielsen, Christian Torp-Pedersen, Regitze Kuhr Skals, Thomas A. Gerds, Zidryne Karaliunaite, Carl-Johan Jakobsen

**Affiliations:** 1 Department of Anesthesia and Intensive Care, Aarhus University Hospital, Palle Juul-Jensens Boulevard, 8200 Aarhus N, Denmark; 2 Department of Health, Science and Technology, Aalborg University, Frederiks Bajersvej, 9220 Aalborg, Denmark; 3 Unit of Epidemiology and Biostatistics, Aalborg University Hospital, Forskningens Hus, Sdr. Skovvej 15, 9000 Aalborg, Denmark; 4 Department of Public Health, Section of Biostatistics, University of Copenhagen, Oester Farimagsgade 5, 1014 Copenhagen, Denmark

**Keywords:** Cardiac surgery, Intraoperative, Milrinone, Dobutamine, Mortality, Retrospective cohort study, g-formula, Standardized mortality risk

## Abstract

**Background:**

Several choices of inotropic therapy are available and used in relation to cardiac surgery. Comparisons are necessary to select optimal therapy. In Denmark, dobutamine and milrinone are the two inotropic agents most commonly used to treat post-bypass low cardiac output syndrome. This study compares all-cause mortality with these drugs.

**Methods:**

In a retrospective observational study we investigated 10,700 consecutive patients undergoing cardiac surgery from 1 April 2006 to 31 December 2013 at Aarhus and Aalborg University Hospitals in the Central and Northern Denmark Region. Prospectively entered data in the Western Danish Heart Registry on intraoperative use of inotropes were used to identify 952 patients treated with milrinone, 418 patients treated with dobutamine, and 82 patients receiving a combination of the two inotropes. All-cause mortality among patients receiving dobutamine was compared to all-cause mortality among milrinone receivers.

Multiple logistic regression analyses including preoperative and intraoperative variables along with g-formula analyses were used to model 30-day and 1-year mortality risks. Reported were standardized mortality risk differences between the treatment groups.

**Results:**

Among patients receiving intraoperative dobutamine, 18 (4.3%) died within 30 days and 49 (11.7%) within 1 year. Corresponding 30-day and 1-year mortality for milrinone receivers were 81 (8.5%) and 170 (17.9%). Risk of death within 30 days and 1 year was increased for intraoperative milrinone compared to dobutamine with a standardized risk difference of 4.06% (confidence interval (CI) 1.23; 6.89, *p* = 0.005) and 4.77% (CI 0.39; 9.15, *p* = 0.033), respectively. Sensitivity analyses including adjustment for milrinone preference, hemodynamic instability prior to cardiopulmonary bypass, and separate analyses on hospital level all confirmed a sign toward increased mortality among milrinone receivers.

**Conclusions:**

Intraoperative use of milrinone in cardiac surgery may be associated with an increase in all-cause mortality compared to use of dobutamine.

**Electronic supplementary material:**

The online version of this article (10.1186/s13054-018-1969-1) contains supplementary material, which is available to authorized users.

## Background

Low cardiac output syndrome is a common complication in cardiac surgery patients, occurring in 3–14% of patients who undergo cardiac surgery with use of cardiopulmonary bypass [1, 2]. Definitions of low cardiac output syndrome vary, but most often include decrease in cardiac index to < 2.0 L/min/m^2^, low systolic blood pressure < 90 mmHg, and signs of tissue hypoperfusion [3]. Inotropic agents form the cornerstone in the perioperative management of low cardiac output syndrome along with mechanical assist device support. Inotropic therapy is known to improve intraoperative hemodynamic variables [4]. However, concern has been raised regarding possible harmful side effects of inotrope treatment such as arrhythmias, increased myocardial oxygen consumption resulting in cardiac ischemia, and potential damage of hibernating but viable myocardium [5, 6]. Thus, despite the apparent immediate clinical improvement achieved with inotrope therapy, there may be a risk of progression of heart failure in patients exposed to inotropes. Noticeably, there is substantial variation in choice of inotropic therapy between cardiac centers and even between individual providers within centers [7, 8], indicating a lack of convincing data on how the most used inotropes compare. Except for recent levosimendan trials [2, 9], in the setting of cardiac surgery not a single randomized clinical trial has ever investigated inotropes/vasopressors using hard outcome parameters including mortality. Hence, data on comparative efficacy or harm associated with individual inotropic drugs are needed. In Denmark, dobutamine and milrinone are the two inotropic agents most commonly used to treat post-bypass low cardiac output syndrome. Differences between milrinone and dobutamine therapy on hemodynamic effects during cardiac surgery have been investigated in only two randomized trials [10, 11]. These trials included 120 and 20 patients, respectively and were not designed to clarify the effects of drugs on mortality and major postoperative complications. Consequently, data on patient outcome following dobutamine versus milrinone treatments have not been subjected to systematic trials and both drugs are commonly used in the same centers. Although meta-analytic data have shown a signal toward increased mortality in cardiac surgery patients randomized to receive milrinone [12], milrinone has become more and more popular among Danish cardiac anesthesiologists. Given the lack of trial data, we performed a large-scale retrospective study of patients treated with intraoperative dobutamine or milrinone during cardiac surgery to compare all-cause mortality at 30 days and after 1 year.

## Methods

### Study design and setting

The study was set up as a two-center retrospective cohort study based on prospectively registered data involving 10,700 consecutive adult patients undergoing cardiac surgery in the Central and Northern Denmark Region at the Aarhus and Aalborg University Hospitals, Denmark. These hospitals provide cardiac surgery for a mixed rural–urban population constituting 33% of the Danish population. The Danish National Health Service provides tax-funded medical care for all Danish residents. This study followed a prespecified analysis plan, including prespecified outcomes, exposures, confounders, and effect modifications and prespecified identification of subgroups for sensitivity analyses. The reporting follows the checklist proposed in the STROBE statement [13].

### Inclusion criteria

Patients older than the age of 14 years undergoing cardiac surgery in the period from 1 April 2006 to 31 December 2013 were included provided they met the following inclusion criteria: coronary artery bypass grafting (CABG), CABG with valve surgery, valve surgery alone or combined with other procedures, or surgery involving the thoracic aorta. Patients with no valid civil registration number, no available data on procedure type, or no information on exposure to inotropes were excluded. Patients were excluded if they had undergone heart transplants, pulmonary thromboendarterectomy, only explorative sternatomy, or surgery for grown-up congenital heart disease. Patients undergoing more than one cardiac surgical procedure during the study period were included with only the last surgical procedure.

**Fig. 1:**
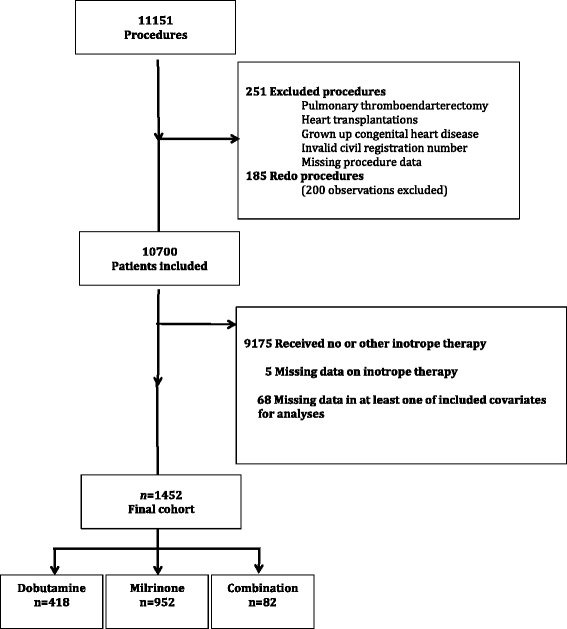
Flowchart of study cohort

Finally, patients receiving no inotrope therapy or inotrope therapy other than dobutamine or milrinone were excluded, leaving 1452 patients for study analyses (Fig. 1).

### Data sources

Data were obtained from the Western Denmark Heart Registry. Registration is mandatory and Internet based, and is completed intraoperatively and postoperatively in the intensive care unit by the surgeons and the attending anesthetists. The registry includes detailed information on patient history, type of procedure, and intraoperative and postoperative management including inotropic therapy and inhospital complications. Data quality is assessed using automatic validation rules at data entry combined with systematic validation procedures and random spot-checks of data after entry. Coverage of the Western Denmark Heart Registry is routinely evaluated by comparison with data from the Danish National Patient Register including data on all procedures performed in both private and public hospitals in Denmark. These analyses have shown a high coverage of the Western Denmark Heart Registry, with more than 95% reporting all CABG procedures. Random samples of the data in the Western Denmark Heart Registry have been validated against local patient files (both electronic and paper files). The quality control of the WDHR has revealed that overall data errors were lower than 3% [14]. The Western Denmark Heart Registry has proven a valuable data source in research, providing ongoing longitudinal registration of detailed data on patients and procedures [15]. Missing data in the registry were retrieved from local patient files (both electronic and paper) and overall missing data in the study cohort constituted less than 0.3% for covariates retrieved from registry and 0% for outcome data.

Registration of intraoperative hemodynamic variables was restricted to Aarhus University Hospital, where data were obtainable from a perioperative electronic database management system. Due to random electronic errors, some hemodynamic variables were lost for registration in approximately one-third of these cases.

### Intraoperative management

All preoperative cardiac medication was continued until the morning of surgery except for angiotensin-converting inhibitors, aspirin, and platelet function inhibitors. Beta-blocking agents were continued on the day of surgery in chronically treated patients. All patients received standard premedication in the form of a benzodiazepine 60–90 min before surgery. Our perioperative management strategies have been described in detail previously [16]. Briefly, all patients received general anesthesia with invasive hemodynamic monitoring, including use of a pulmonary artery catheter (standard Swan-Ganz catheter; Edwards Lifesciences, Irvine, CA, USA) with or without continuous cardiac output measurements and transesophageal echocardiography. The standardized cardiopulmonary bypass (CPB) technique included use of a closed system with a modified additive coating system and a venous reservoir, priming with crystalloid, and routine blood flow rates at 2.4 L/min/m^2^ aiming at a mean arterial pressure of 50–60 mmHg. Patients were maintained norm thermic or mildly hypothermic. Myocardial protection was achieved by either intermittent antegrade cold crystalloid or blood cardioplegia with 20-min intervals. Cardioplegia was given retrograde when appropriate. At the end of the surgical procedure, reperfusion of the heart was performed on an individual basis according to the patient’s general condition and time on cross clamp. Use of calcium at the termination of CPB was at the discretion of the attending anesthesiologist. At the end of surgery, patients were transferred to the intensive care unit (ICU). There was no fixed postoperative treatment regimen for either pharmaceutical or mechanical support. Patients were extubated when they were hemodynamic stable and with no need of respiratory support. Following routine practice, patients were discharged from the ICU to the general ward within the first postoperative day, on condition of being clinically stable and with no need for hemodynamic or respiratory support.

### Inotropic treatment

Neither institutional guidelines nor specified algorithms dictating intraoperative inotropic therapy were used in the participating centers. Consequently, the intraoperative strategy of inotrope use and discontinuation of inotrope use were at the discretion of the attending anesthetist. However, if inotrope treatment was considered necessary, the standard procedure in both centers was to initiate inotrope treatment at the end of, or after CPB. Patients who had received dobutamine in any dosage intraoperatively either as single therapy or with concurrent vasoconstrictor infusion were classified as exposed to “intraoperative dobutamine”. Patients who had received milrinone administered as infusion or/and as bolus while on CPB with or without concurrent vasoconstrictor infusion were classified as exposed to “intraoperative milrinone”. Patients who had received both inotropes during operation were classified as “intraoperative combination”. Data on intraoperative inotrope therapy was entered prospectively in the Western Danish Hearth Registry.

### Primary outcome measure

The primary outcomes of this study were 30-day and 1-year all-cause mortality. Date of death was obtained from the Civil Registration System [17]. All patients in the study cohort had at least 1 year of follow-up after discharge from the ICU. None of the 1452 patients included in the analyses presented missing data on mortality outcomes.

### Confounders

In an earlier study, we identified patient and procedural factors associated with use of inotropic therapy in cardiac surgery [7]. These potential confounding factors were collected through the Western Danish Heart Registry and included the additive EuroSCORE I [18, 19], type of cardiac procedure, time on CPB, attending anesthetist preference of inotrope drug, cardiac center, anesthetic technique, and year of surgery. Left ventricular function and type of surgery might strongly influence decision-making with respect to inotrope use, and weighting in the EuroSCORE score might not reflect this; consequently, these variables were analyzed separately. To avoid including confounding factors twice (both as single parameters and in the EuroSCORE), a modified version of the EuroSCORE was designed, excluding gender, age, left ventricular function, and type of cardiac surgery. The modified EuroSCORE with included variables and scoring is presented in Additional file 1: Table S1. Cardiac procedures were separated into three groups: CABG-only group, aortic valve repair or replacement only or in combination with other procedure group, and mitral valve repair or replacement only or in combination with other procedure group. Time on cardiopulmonary bypass was grouped as offpump, less than or equal to 120 min, or exceeding 120 min. Anesthetic techniques were expressed as intravenous anesthesia or volatile anesthesia. When available, the precardiopulmonary cardiac index (L/min/m^2^) and mixed venous oxygen saturation were included as confounders. A mean of four consecutive measurements every 15 min within 1 h prior to cardiopulmonary bypass was included for analyses.

### Statistical methods

Preoperation patient characteristics were summarized according to the treatment groups (intraoperative milrinone, intraoperative dobutamine, and intraoperative combination therapy), and compared across treatment groups using chi-square tests for categorical variables and analysis of variance for continuous variables. Crude 30-day and 1-year mortality were computed and reported as relative frequencies. Patient characteristics and procedural factors may confound a direct comparison of relative mortality frequencies between patients receiving milrinone compared to dobutamine receivers. Therefore, the main analysis was based on a multiple logistic regression model for 30-day and 1-year mortality outcome according to milrinone versus dobutamine treatment or combination therapy and further adjusted for age (< 60 years, 60–69 years, 70–75 years, > 75 years), gender, left ventricular ejection fraction (≤ 30%, 31–50%, > 51%), cardiac procedure type, time on cardiopulmonary bypass (off-pump, ≤ 120 min, > 120 min), type of anesthesia, year of surgery, and modified total EuroSCORE.

To enable a clinical interpretation, causal inference methodology was implemented [20].

Theoretically, a detrimental effect of one of the drugs could be masked by patients having a beneficial effect of the other drug received postoperatively. Such an effect would result in the intraoperative combination having a lower mortality than at least one of the drugs used alone. Accordingly, combination therapy with both dobutamine and milrinone was included in the data analysis. Because there were three groups of treatment, propensity matching was not used and the g-formula model was used instead. The g-formula model uses the derived logistic model to predict outcome for the whole population for each of the three treatment groups, thereby ensuring identical covariate status for the comparison. The derived differences in mortality are thereby adjusted for all confounders, but still depend on the model being appropriate and the absence of unmeasured confounding.

From the logistic regression analysis, we computed three standardized mortality risks for each patient using the g-formula approach [21–23]. With “standardized mortality risks”, we refer to the probability that a patient with a given combination of risk factors dies within 30 days or within 1 year according to our logistic regression model. For the first (second, third) standardized mortality risks, we predicted the probability of 30-day or 1-year mortality conditioning on actual values of the confounders but assuming possible counter to the fact that all patients received dobutamine (milrinone, combination therapy). Reported were differences in averages of standardized 30-day and 1-year mortality risks between dobutamine versus milrinone treatment or combination therapy. Confidence intervals and Wald tests for the standardized risk differences were obtained using bootstrap standard errors based on 5000 bootstrap samples.

We included milrinone preference as an additional confounder to determine whether there was an influence if the attending anesthetist had a preference for using milrinone as inotropic therapy. Only anesthetists who had been attending surgeries during the entire study period were included for this analysis, leaving 872 patients for analysis. Preference status was based on frequency of milrinone use in the first half of study period, grouping each anesthetist into quartiles: use of milrinone for less than 48% of his/her patients, 48–70% of his/her patients, 71–83% of his/her patients, or more than 84% of his/her patients in this baseline period. Accordingly, the numbers in each treatment group refer to the numbers of patients anesthetized by doctors assigned to each of the four quartiles of milrinone preference.

Further, to determine whether the hemodynamic status immediately before initiating cardiopulmonary bypass modified the results, a sensitivity analysis was performed in the subset of patients with available data on hemodynamic variables. Additional sensitivity analyses included separate analyses on patients with surgery performed in the same hospital.

Treatment effect modification was investigated regarding age groups, gender, period, and type of surgery (CABG, aortic valve repair, or mitral valve repair).

All *p* values were two-tailed, and *p* < 0.05 was considered statistically significant.

The number of cases in the registry during the study period defined the sample size.

Analyses were all performed using the statistical software R version 3.3.1. [24].

## Results

Crude 30-day and 1-year mortality risks after admission to the ICU for all 10,700 patients included in study were 1.9% (117/10,700) and 2.3% (250/10,700), respectively. The final cohort for analysis of intraoperative inotrope treatment consisted of 1452 patients.

Preoperation characteristics across treatment for these patients are presented in Table 1, omitting 82 patients receiving combination therapy. Being older, with lower preoperative ejection fraction, higher EuroSCORE, and longer time on cardiopulmonary bypass, and having more complex surgery characterized patients receiving milrinone.

**Table 1:** Baseline characteristics

Variable	Dobutamine (*n* = 418)	Milrinone (*n* = 952)	*p* value
Age group (years)			
< 60	77 (18.4)	189 (19.9)	
60–69	122 (29.2)	262 (27.5)	
70–75	92 (22.0)	236 (24.8)	
> 75	127 (30.4)	265 (27.8)	0.53
Gender			
Female	138 (33.0)	253 (26.6)	0.02
LVEF			
> 50%	189 (45.2)	347 (36.4)	
30–50%	165 (39.5)	324 (34.0)	
< 30%	64 (15.3)	281 (29.5)	< 0.01
Modified EuroSCORE, mean (SD)	1.6 (1.5)	1.7 (1.5)	0.38
Time on CPB			
Offpump	55 (13.2)	23 (2.4)	
< 120 min	184 (44.0)	316 (33.2)	
> 120 min	179 (42.8)	613 (64.4)	< 0.01
Anesthesia			
Intravenous	315 (75.4)	400 (42.0)	
Volatile	103 (24.6)	552 (58)	< 0.01
Year period			
2006–2008	173 (41.4)	296 (31.1)	
2009–2011	158 (37.8)	389 (40.9)	
2012–2013	87 (20.8)	267 (28.0)	< 0.01
Hospital			
Aarhus UH	327 (78.2)	399 (41.9)	
Aalborg UH	91 (21.8)	553 (58.1)	< 0.01
Type of surgery			
CABG only	143 (34.2)	277 (29.1)	0.07
Mitral valve	93 (22.2)	221 (23.2)	0.75
Aortic	125 (29.9)	352 (37.1)	0.01
Known milrinone preference^a^ (% of procedures)			
< 48%	103 (45.0)	107 (19.9)	
48–70%	65 (28.4)	113 (21.0)	
70–83%	29 (12.7)	128 (23.8)	
> 83%	32 (14.0)	189 (35.2)	0.01
Cardiac index (L/min/m^2^)			
≤ 2	105 (50.5)	137 (50.6)	1.00
S_v_O_2_			
≤ 60	30 (14.4)	51 (18.8)	0.25
Other intraoperative vasoactive support			
Norepinephrine	196 (46.9)	719 (75.5)	< 0.01
Epinephrine	13 (3.1)	114 (12.0)	< 0.01
Dopamine	5 (1.2)	16 (1.7)	0.01
Levosimendan	7 (1.7)	1 (0.1)	< 0.01
Postoperative mechanical support			
ECMO	6 (1.4)	12 (1.3)	0.84
IABP	15 (3.6)	118 (12.4)	< 0.01

Baseline characteristics according to intraoperative inotrope treatment. Eighty-two patients in the mixture group were omitted. Data presented as *n* (%) unless stated otherwise

*LVEF* left ventricular ejection fraction, *SD* standard deviation, *CPB* cardiopulmonary bypass, *UH* University Hospital, *CABG* coronary artery bypass grafting, *SvO*_*2*_ mixed venous oxygen saturation, *ECMO* extracorporal membrane oxygenation, *IABP* intra-aortic balloon pump

^a^Attending anesthetist’s preference for milrinone was established as a covariate stratified according to fraction of prior surgeries in which the attending anesthetist used milrinone

Crude risks of 30-day and 1-year mortality for patients receiving intraoperative dobutamine were 4.3% (18/418) and 11.7% (49/418) respectively. Corresponding 30-day and 1-year mortality risks for milrinone receivers were 8.5% (81/952) and 17.9% (170/952), and for patients receiving combination therapy were 22% (18/82) and 37.8% (31/82).

Having excluded a theoretical beneficial effect of combination therapy, figures and tables include only estimates for milrinone versus dobutamine therapy.

**Fig. 2:**
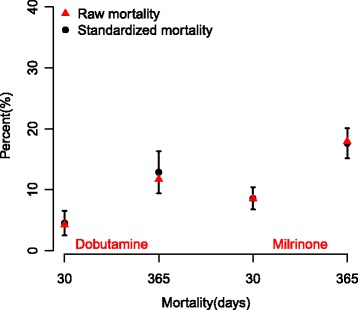
Raw and standardized 30-day and 1-year mortality according to inotrope treatment

Figure 2 shows these crude mortality risks as well as standardized mortality risks by intraoperative treatment.

Table 2 presents the differences in standardized 30-day and 1-year mortality according to intraoperative inotrope treatment obtained from the main analysis and from sensitivity analyses.

**Table 2:** Difference in standardized mortality rates according to intraoperative inotrope treatment

Statistical model	Number of patients	Standardized risk difference (%)(95% CI)	*p* value
Main analyses			
Crude 30-day mortality, milrinone vs dobutamine	1452	4.2 (1.6; 6.8)	< 0.01
Adjusted 30-day mortality, milrinone vs dobutamine	1452	4.1 (1.2; 6.9)	< 0.01
Crude 1-year mortality, milrinone vs dobutamine	1452	6.1 (2.2; 10.1)	< 0.01
Adjusted 1-year mortality, milrinone vs dobutamine	1452	4.8 (0.4; 9.2)	0.03
Sensitivity analyses on subpopulations			
Adjustment for anesthetist’s preference of milrinone			
Crude 30-day mortality, milrinone vs dobutamine	817	7.4 (4.4; 10.3)	< 0.01
Adjusted 30-day mortality, milrinone vs dobutamine	817	6.2 (2.7; 9.6)	< 0.01
Adjustment for hemodynamic status prior to cardiopulmonary bypass			
Crude 30-day mortality, milrinone vs dobutamine	533	2.1 (−2.2; 6.4)	0.34
Adjusted 30-day mortality, milrinone vs dobutamine	533	1.4 (−3.6; 6.4)	0.59
Centers analyzed separately			
Crude 30-day mortality Aarhus, milrinone vs dobutamine	726	3.4 (−0.1; 6.9)	0.06
Adjusted 30-day mortality Aarhus, milrinone vs dobutamine	726	3.7 (−0.2; 7.5)	0.06
Crude 30-day mortality Aalborg, milrinone vs dobutamine	644	6.5 (2.7; 10.3)	< 0.01
Adjusted 30-day mortality Aalborg, milrinone vs dobutamine	644	6.2 (2.3; 10.2)	< 0.01

CI confidence interval

Treatment with milrinone was associated with standardized 30-day mortality and 1-year mortality risk differences of 4.1% (CI 1.2; 6.9, *p* < 0.01) and 4.8% (CI 0.4; 9.2, *p* = 0.03), respectively. Corresponding adjusted standardized mortality for combination therapy was 13.2% (CI 5.4; 21.0, *p* < 0.01) and 18.5% (CI 8.3; 28.8).

A total of 533 patients undergoing surgery at Aarhus University Hospital had information on cardiac index and central venous saturation prior to cardiopulmonary bypass. Including these hemodynamic data in the regression model, no significant difference between treatment groups was evident (*p* = 0.60) (Table 2).

Although insignificant, sensitivity analyses analyzing centers separately indicated a tendency toward increased mortality associated with milrinone use in both centers (Table 2).

**Fig. 3:**
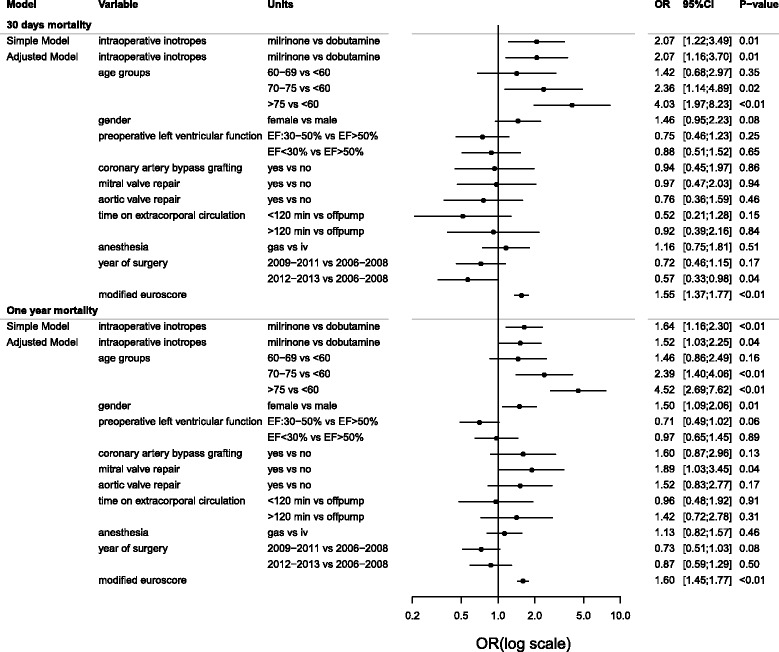
Crude and adjusted odds ratios of 30-day and 1-year mortality according to inotrope regime. Simple model, unadjusted estimates; adjusted model, estimates adjusted for listed covariates. Modified EuroSCORE is score on preoperative condition of chronic pulmonary disease, extracardiac arteriopathy, neurological dysfunction, previous cardiac surgery, baseline plasma creatinine, active endocarditis, critical preoperative state, emergency, recent myocardial infarction, pulmonary hypertension, and postinfarct septal rupture. OR odds ratio, CI confidence interval, EF preoperative left ventricular ejection fraction, iv intravenous

According to the multiple logistic regression analysis, older age, EuroSCORE, and surgery performed late in the study period were also found to be independently associated with an increased 30-day mortality risk, whereas female gender, mitral valve surgery, and EuroSCORE were found associated with increased 1-year mortality risk (Fig. 3).

Hemodynamic profiles in treatment groups were comparable in the immediate precardiopulmonary and postcardiopulmonary bypass periods, except for milrinone receivers presenting with lower mean arterial blood pressure. Hemodynamic variables are presented in Additional file 2: Table S2.

## Discussion

This retrospective study found intraoperative milrinone treatment to be associated with an increased mortality compared with dobutamine treatment in cardiac surgery patients. The main analysis was supplemented with a number of sensitivity analyses all confirming a tendency toward increased mortality among milrinone receivers. A few observational studies have reported increased mortality associated with overall inotrope use in the cardiac surgery setting [16, 25]. However, inconsistent data support the superiority of one inotropic therapy over the other. Accordingly, choice of inotropes in the treatment of cardiac failure both in a post-bypass situation and in nonsurgical heart failure seem to be largely guided by experts’ opinion along with institutional and provider preference [26–29]. In Denmark, use of milrinone for cardiac failure following cardiopulmonary bypass has increased at the expense of dobutamine use. Milrinone was introduced as an agent which, compared to dobutamine, cause reduced left and right heart filling pressures due to its greater reduction in vascular resistance, and thus superior to dobutamine in treatment of low cardiac output syndrome following cardiac surgery [30, 31]. However, in reviewing the literature, we found only two randomized trials comparing the isolated action of milrinone versus dobutamine in cardiac surgery. The notion that milrinone is superior to dobutamine seems to be based on one randomized trial including 120 patients randomized to either bolus and infusion milrinone or infusion dobutamine (60 patients in each treatment arm) if meeting an entry criterion of a cardiac index < 2.0 L/min/m^2^ within 2 h after separation from cardiopulmonary bypass [10]. Both drugs were found equally effective to reverse low cardiac output after cardiac surgery, and in subgroup analysis were equally effective at treating pulmonary hypertension. More adverse events were noted in the dobutamine arm with a higher incidence of hypertension and atrial fibrillation, whereas milrinone was associated with a higher incidence of bradycardia. The study was not powered to estimate effect on mortality and no long-term postoperative outcomes were reported. The only other study, a small randomized study among 20 patients undergoing cardiac surgery, however, found no marked differences in hemodynamic parameters according to treatment with dobutamine or milrinone [11]. The study provided no information on clinical outcomes. Earlier trials comparing the efficacy of early-generation phosphodiesterase inhibitors (maranon and enoximone) with dobutamine similarly lack data on effect on major clinical outcomes [4]. Recent meta-analyses of randomized clinical trials with milrinone for cardiac dysfunction conclude that the use of milrinone is neither to be recommended nor refuted due to risk of bias and random error in current evidence. None of the included studies in these meta-analyses used dobutamine as a single comparator [32, 33]. One study using network meta-analytic data including indirect comparisons suggested no big differences between milrinone and dobutamine in cardiac surgery [34]. To date, the only randomized trial sufficiently powered to establish the safety and efficacy profile of milrinone was the OPTIME-CHF trial enrolling 951 patients with nonsurgical acute exacerbation of chronic heart failure. The results of this trial suggested that milrinone might be harmful in patients with ischemic heart failure with LVEF < 40%. However, data are difficult to interpret as the study allowed cointerventions with dobutamine in randomized patients [35, 36].

Given the paucity of data from randomized trials, the present observational study raises a highly relevant clinical question regarding the perceived “superiority” of milrinone compared to dobutamine as intraoperative treatment for low cardiac output syndrome following cardiopulmonary bypass. Particularly, in the light of results from the recent levosimendan trials suggesting no major clinical improvement using levosimendan in cardiac surgery [2, 9], superiority of clinically relevant outcomes between “old” inotropes remains an important clinical question.

The observed increased mortality associated with use of milrinone might be associated with the potent vasodilator effect of milrinone. Use of intravenous milrinone is known to be associated with systemic hypotension, and the safety margin could be significantly reduced if volume loading is not optimized or patients are already on vasopressor therapy [30, 37, 38]. Milrinone is often given on a preemptive basis for patients with preoperative low EF. However, the dosage and safety of preemptive milrinone have never been uniformly established [39, 40]. Going off bypass is a period with huge changes in cardiac filling pressures and milrinone loading could induce severe hypotension, especially in patients already on vasopressor therapy while on bypass. Vasoplegia following cardiac surgery is a serious condition, implying a high risk of postoperative organ failure and increased mortality [41, 42]. Clinicians may tend to perceive milrinone primarily as a potent inotrope, whereas several studies indicate that milrinone is mainly a potent vasodilator, with limited inotrope properties against a fixed afterload [43, 44]. Thus, theoretical advantages related to the afterload-reducing effects may be diminished by the need for vasopressor therapy [4]. Recent updates on inotropes for cardiac patients recommend that dobutamine is preferable in clinical states characterized by hypotension compared with milrinone [45].

Approximately one-third of patients in the present study comprised patients with ischemic heart disease. Accordingly, in reference to the results from the OPTIME-CHF trial [36], this could additionally explain the observed increased mortality associated with the use of milrinone.

Use of milrinone has been considered particularly relevant for patients with diastolic dysfunction or pulmonary hypertension, but two trials challenge these notions. Couture et al. [46] randomized 50 patients undergoing CABG to receive milrinone or placebo starting before CPB and continuing until skin closure. They found no improvement in biventricular diastolic function. The effects were only observed on systolic function. Denault et al. [47] evaluated the efficacy of inhaled milrinone for pulmonary hypertension and right ventricular dysfunction on clinically relevant endpoints following high-risk cardiac surgery patients in a recent study. Patients (*N* = 124) were randomized to inhaled milrinone or placebo if presenting with preoperative pulmonary hypertension (mean pulmonary artery pressure > 30 mmHg or systolic pulmonary pressure > 40 mmHg). Despite favorable hemodynamic effects in the milrinone arm, milrinone did not facilitate separation from cardiopulmonary bypass nor prevent right ventricular failure. Accordingly, one could speculate that clinicians may overvalue the effects of milrinone in these special settings, and overlook the risk of the vasodilation effects of milrinone in patients already presenting with vasopressor dependency. Another comment should be given on the dosage of dobutamine; commonly reported dosages from older studies range from 10 to 20 μg/kg/min [10]. Our current practice includes dobutamine doses in the range of 5–8 μg/kg/min. Last but not least, milrinone is eliminated via the kidneys and infusion should be adjusted in patients with impaired kidney function. Unfortunately, exact dosages of either inotropes were unavailable in our study.

The low proportion of patients receiving inotropic therapy (14%) indicates that inotropes were administered not on a routine basis, but to the sickest patients. However, data did not allow determining whether inotropes were used driven by a firm diagnosis of low cardiac output syndrome. Data do not unveil the sequence of inotrope treatment and hemodynamic status. Given this limitation, however, hemodynamic variables might indicate that only a smaller proportion of inotrope receivers suffered from postcardiopulmonary low cardiac output syndrome (Additional file 2: Table S2).

We cannot completely exclude that adjusted mortality is influenced by differences in disease severity, but sensitivity analyses including adjustment for precardiopulmonary hemodynamic status aimed to ensure that treatment groups were comparable before going on bypass. Further, patients’ hemodynamic profiles were also comparable between treatment groups in the immediate postcardiopulmonary period, except for milrinone receivers presenting with lower mean arterial blood pressure.

The present study illustrates that “new” drugs should not be administered before large, pragmatic, high-quality multicenter randomized controlled trials have been performed. Proof of benefit and safety profiles between dobutamine and milrinone have never been properly characterized, as both catecholamines and phosphodiesterase inhibitors were established before long-term outcome testing became mandatory. Thus, the present study adds important information to the existing literature on the topic.

Our study has several strengths. It is the largest study to date to compare mortality associated with the use of the two most commonly used inotropes in Denmark. Further, the strengths of this study include the population-based nature of the cohort, with complete long-term follow-up ensured by the unique personal identification number. Included confounders were defined based on the findings of a prior study identifying preoperative and intraoperative factors associated with the use of inotrope therapy [7]. Including these confounders into the g-formula, adjustment was performed to control for indication bias. The main limitation of the study is its observational nature. The study shows associations, but cannot prove causality. As with any statistical analysis, our ability to adjust for potential confounding is limited to available data. By performing analyses including the patient’s hemodynamic profile prior to inotrope exposure and the anesthetists’ preferences of inotrope, attempts were made to adjust for possible preference for one therapy over another, and for patients with increased cardiac disease burden. We recognize is a major limitation that 1/3 of cases have missing data on their hemodynamic profile. However, hemodynamic data were considered missing at random, as no systematic error could explain why data had not been registered electronically. In terms of understanding the indications for use of inotropes and potential benefits or risks, the available hemodynamic data were considered highly valuable and appropriate for subgroup analysis. Preferably, the analyses should also have included echocardiographic evaluations made at the initiation of inotropic therapy. Unfortunately, echocardiographic reports were not part of the standard registration procedure in either hospital. Consequently, echocardiographic data were not available. Hence, we cannot rule out residual confounding of worse cardiac performance among milrinone receivers, relating to intraoperative factors such as reperfusion injury or cardioplegia-induced myocardial dysfunction not necessarily accounted for by procedure scoring. Use of vasoconstrictors was more common in the milrinone group, suggesting that milrinone receivers were more hemodynamically unstable than patients receiving dobutamine. Unfortunately, the data did not allow establishing the sequence or dosage of other vasoactive therapy. Accordingly, it is not possible to determine whether more frequent use of norepinephrine is to be explained by hypotension caused by milrinone infusion, or if milrinone was used as second-line inotrope therapy. Similar, the more frequent use of postoperative mechanical support among milrinone receivers suggests that these patients were more hemodynamically unstable than their comparisons, and that unmeasured confounding could bias results despite adequate adjustment methods. None of the participating centers followed a strict goal-directed therapy strategy regarding the dosage of inotrope therapy, and it could be discussed whether inotropes were administered in adequate dosages and started and stopped appropriately. Neither did data allow for subgroup analyses of a beneficial effect of milrinone in patients on chronic beta-blocker treatment.

## Conclusion

The retrospective data reported here indicate a possible increased risk of mortality associated with the use of intraoperative milrinone compared with use of dobutamine among cardiac surgery patients. It is important to bear in mind the possible bias due to the nature of the data. However, the present results highlight the need for a large-scale randomized trial to reevaluate possible harms and benefits associated with the two most commonly used inotropic agents used for low cardiac output syndrome in cardiac surgery.

## Additional files

Additional file 1: **Table S1.** presenting definitions and scores of covariates included in the modified EuroSCORE (DOCX 17 kb)

Additional file 2: **Table S2.** presenting hemodynamic variables according to inotrope therapy pre and post CPB (DOCX 20 kb)

## Abbreviations

CABG: Coronary artery bypass grafting
CI: Confidence interval
CPB: Cardiopulmonary bypass
ECMO: Extracorporal membrane oxygenation
IABP: Intra-aortic balloon pump
ICU: Intensive care unit
LVEF: Preoperative left ventricular ejection fraction
S_v_O_2_: Central venous oxygenation

## References

[1] Algarni KD, Maganti M, Yau TM (2011). Predictors of low cardiac output syndrome after isolated coronary artery bypass surgery: trends over 20 years. Ann Thorac Surg.

[2] Landoni G, Lomivorotov VV, Alvaro G (2017). Levosimendan for hemodynamic support after cardiac surgery. N Engl J Med.

[3] Lomivorotov VV, Efremov SM, Kirov MY, Fominskiy EV, Karaskov AM (2017). Low-cardiac-output syndrome after cardiac surgery. J Cardiothorac Vasc Anesth.

[4] Gillies M, Bellomo R, Doolan L, Buxton B (2005). Bench-to-bedside review: Inotropic drug therapy after adult cardiac surgery—a systematic literature review. Crit Care.

[5] Indolfi C, Piscione F, Perrone-Filardi P (1996). Inotropic stimulation by dobutamine increases left ventricular regional function at the expense of metabolism in hibernating myocardium. Am Heart J.

[6] Schulz R, Rose J, Martin C, Brodde OE, Heusch G (1993). Development of short-term myocardial hibernation. its limitation by the severity of ischemia and inotropic stimulation. Circulation.

[7] Nielsen DV, Johnsen SP, Madsen M, Jakobsen CJ (2011). Variation in use of peroperative inotropic support therapy in cardiac surgery: time for reflection?. Acta Anaesthesiol Scand.

[8] Hernandez AF, Li S, Dokholyan RS, O'Brien SM, Ferguson TB, Peterson ED (2009). Variation in perioperative vasoactive therapy in cardiovascular surgical care: data from the society of thoracic surgeons. Am Heart J.

[9] Mehta RH, Leimberger JD, van Diepen S (2017). Levosimendan in patients with left ventricular dysfunction undergoing cardiac surgery. N Engl J Med.

[10] Feneck RO, Sherry KM, Withington PS (2001). Oduro-Dominah A, European Milrinone Multicenter Trial Group. Comparison of the hemodynamic effects of milrinone with dobutamine in patients after cardiac surgery. J Cardiothorac Vasc Anesth.

[11] Carmona MJ, Martins LM, Vane MF, Longo BA, Paredes LS, Malbouisson LM (2010). Comparison of the effects of dobutamine and milrinone on hemodynamic parameters and oxygen supply in patients undergoing cardiac surgery with low cardiac output after anesthetic induction. Rev Bras Anestesiol.

[12] Zangrillo A, Biondi-Zoccai G, Ponschab M (2012). Milrinone and mortality in adult cardiac surgery: a meta-analysis. J Cardiothorac Vasc Anesth.

[13] Vandenbroucke JP, von Elm E, Altman DG (2007). Strengthening the reporting of observational studies in epidemiology (STROBE): explanation and elaboration. Epidemiology.

[14] Rasmussen LA, Botker HE, Jensen LO (2017). Quality assurance of the western denmark heart registry, a population-based healthcare register. Dan Med J.

[15] Schmidt M, Maeng M, Jakobsen CJ (2010). Existing data sources for clinical epidemiology: the Western Denmark Heart Registry. Clin Epidemiol.

[16] Nielsen DV, Hansen MK, Johnsen SP, Hansen M, Hindsholm K, Jakobsen CJ (2014). Health outcomes with and without use of inotropic therapy in cardiac surgery: results of a propensity score-matched analysis. Anesthesiology.

[17] Pedersen CB, Gotzsche H, Moller JO, Mortensen PB (2006). The Danish civil registration system. A cohort of eight million persons. Dan Med Bull.

[18] Nashef SA, Roques F, Michel P, Gauducheau E, Lemeshow S, Salamon R (1999). European system for cardiac operative risk evaluation (EuroSCORE). Eur J Cardiothorac Surg.

[19] Roques F, Nashef SA, Michel P (1999). Risk factors and outcome in european cardiac surgery: analysis of the EuroSCORE multinational database of 19030 patients. Eur J Cardiothorac Surg.

[20] Wang A, Nianogo RA, Arah OA (2017). G-computation of average treatment effects on the treated and the untreated. BMC Med Res Methodol.

[21] Robins J (1986). A new approach to causal inference in mortality studies with a sustained exposure period—application to control of the healthy worker survivor effect. Math Model.

[22] Pearl J (2009). Causal inference in statistics: an overview. Stat Sur.

[23] Hernan M. Causal inference book. 2016. https://www.hsph.harvard.edu/miguel-hernan/causal-inference-book/. Accessed 6 Oct 2017.

[24] R Core Team. R: a language and environment for statistical computing. 2015. https://www.r-project.org. Accessed 6 Oct 2017.

[25] Shahin J, Devarennes B, Tse CW, Amarica DA, Dial S (2011). The relationship between inotrope exposure, six-hour postoperative physiological variables, hospital mortality and renal dysfunction in patients undergoing cardiac surgery. Crit Care.

[26] Allen LA, Fonarow GC, Grau-Sepulveda MV (2014). Hospital variation in intravenous inotrope use for patients hospitalized with heart failure: insights from get with the guidelines. Circ Heart Fail.

[27] Yancy CW, Jessup M, Writing committee members (2013). 2013 ACCF/AHA guideline for the management of heart failure: a report of the American College of Cardiology Foundation/american Heart Association Task Force on practice guidelines. Circulation.

[28] Butterworth JF, Legault C, Royster RL, Hammon JW (1998). Factors that predict the use of positive inotropic drug support after cardiac valve surgery. Anesth Analg.

[29] Mebazaa A, Pitsis AA, Rudiger A (2010). Clinical review: Practical recommendations on the management of perioperative heart failure in cardiac surgery. Crit Care.

[30] Colucci WS, Wright RF, Jaski BE, Fifer MA, Braunwald E (1986). Milrinone and dobutamine in severe heart failure: differing hemodynamic effects and individual patient responsiveness. Circulation.

[31] Wright EM, Sherry KM (1991). Clinical and haemodynamic effects of milrinone in the treatment of low cardiac output after cardiac surgery. Br J Anaesth.

[32] Ushio M, Egi M, Wakabayashi J (2016). Impact of milrinone administration in adult cardiac surgery patients: updated meta-analysis. J Cardiothorac Vasc Anesth.

[33] Koster G, Bekema HJ, Wetterslev J, Gluud C, Keus F, van der Horst IC (2016). Milrinone for cardiac dysfunction in critically ill adult patients: a systematic review of randomised clinical trials with meta-analysis and trial sequential analysis. Intensive Care Med.

[34] Greco T, Calabro MG, Covello RD (2015). A Bayesian network meta-analysis on the effect of inodilatory agents on mortality. Br J Anaesth.

[35] Cuffe MS, Califf RM, Adams KF (2002). Short-term intravenous milrinone for acute exacerbation of chronic heart failure: a randomized controlled trial. JAMA.

[36] Felker GM, Benza RL, Chandler AB (2003). Heart failure etiology and response to milrinone in decompensated heart failure: results from the OPTIME-CHF study. J Am Coll Cardiol.

[37] Feneck RO (1992). Intravenous milrinone following cardiac surgery: I. Effects of bolus infusion followed by variable dose maintenance infusion. the European Milrinone Multicentre Trial Group. J Cardiothorac Vasc Anesth.

[38] Ludmer PL, Wright RF, Arnold JM, Ganz P, Braunwald E, Colucci WS (1986). Separation of the direct myocardial and vasodilator actions of milrinone administered by an intracoronary infusion technique. Circulation.

[39] Arbeus M, Axelsson B, Friberg O, Magnuson A, Bodin L, Hultman J (2009). Milrinone increases flow in coronary artery bypass grafts after cardiopulmonary bypass: a prospective, randomized, double-blind, placebo-controlled study. J Cardiothorac Vasc Anesth.

[40] Oztekin I, Yazici S, Oztekin DS, Goksel O, Issever H, Canik S (2007). Effects of low-dose milrinone on weaning from cardiopulmonary bypass and after in patients with mitral stenosis and pulmonary hypertension. Yakugaku Zasshi.

[41] Mekontso-Dessap A, Houel R, Soustelle C, Kirsch M, Thebert D, Loisance DY (2001). Risk factors for post-cardiopulmonary bypass vasoplegia in patients with preserved left ventricular function. Ann Thorac Surg.

[42] Weis F, Kilger E, Beiras-Fernandez A (2006). Association between vasopressor dependence and early outcome in patients after cardiac surgery. Anaesthesia.

[43] DeWitt ES, Black KJ, Thiagarajan RR (2016). Effects of commonly used inotropes on myocardial function and oxygen consumption under constant ventricular loading conditions. J Appl Physiol.

[44] Eichhorn EJ, Konstam MA, Weiland DS (1987). Differential effects of milrinone and dobutamine on right ventricular preload, afterload and systolic performance in congestive heart failure secondary to ischemic or idiopathic dilated cardiomyopathy. Am J Cardiol.

[45] Parissis JT, Rafouli-Stergiou P, Stasinos V, Psarogiannakopoulos P, Mebazaa A (2010). Inotropes in cardiac patients: update 2011. Curr Opin Crit Care.

[46] Couture P, Denault AY, Pellerin M, Tardif JC (2007). Milrinone enhances systolic, but not diastolic function during coronary artery bypass grafting surgery. Can J Anaesth.

[47] Denault AY, Bussieres JS, Arellano R (2016). A multicentre randomized-controlled trial of inhaled milrinone in high-risk cardiac surgical patients. Can J Anaesth.

