# Supplementary material for: Intraoperative milrinone versus dobutamine in cardiac surgery patients: a retrospective cohort study on mortality
Source: Crit Care. 2018 Feb 26;22:51. doi: 10.1186/s13054-018-1969-1 (PMC5828330; doi:10.1186/s13054-018-1969-1)
Supplement: Supplementary file 1 — Table S1. presenting definitions and scores of covariates included in the modified EuroSCORE (DOCX 17 kb) [file 13054_2018_1969_MOESM1_ESM.docx]

| **Additional Table S1. Definitions and scores of covariates included in the modified EuroSCORE** | | | | | |
| --- | --- | --- | --- | --- | --- |
| **Covariate** | Definition | Score | | Dobutamine | Milrinone |
|  |  |  |  | n=418  (%) | n=952  (%) |
| Chronic pulmonary disease | Long-term use of bronchodilators or steroids for lung disease. | | 1 | 77 (20) | 77 (17.4) |
| Extra cardiac arteriopathy | Any one or more of the following: claudication, carotid occlusion or >50% stenosis, previous or planned intervention on the abdominal aorta, limb arteries, or carotids. | | 2 | 49 (12.7) | 32 (7.2) |
| Neurological dysfunction | Disease severely affecting ambulation or day-to-day functioning. | | 2 | 35 (9.0) | 120 (8.2) |
| Previous cardiac surgery | Requiring opening of the pericardium. | | 3 | 57 (14.5) | 76 (16.9) |
| Baseline plasma creatinine | >200 µmol/L preoperatively. | | 2 | 24 (6.1) | 35 (7.8) |
| Active endocarditis | Patient still under antibiotic treatment for endocarditis at the time of surgery. | | 3 | 24 (6.1) | 30 (6.7) |
| Critical preoperative state | Any one or more of the following: ventricular tachycardia, or fibrillation, or aborted sudden death, preoperative cardiac massage, preoperative ventilation before arrival in the anesthetic room, preoperative inotropic support, intraaortic balloon counterpulsation or preoperative acute renal failure (anuria or oliguria < 10 ml/h). | | 3 | 61 (15.9) | 97 (21.5) |
| Emergency | Carried out on referral before the beginning of the next working day | | 2 | 74 (18.8) | 86 (19.1) |
| Recent myocardial infarction | < 90 days | | 2 | 109 (28.7) | 111 (25.2) |
| Pulmonary hypertension  Post infarct septal rupture | Systolic pulmonary artery pressure >60 mmHg. | | 2  4 | 86 (22.4)  4 (1.0) | 100 (22.8)  4(0.9) |
|  |  | |  |  |  |
|  |  | |  |  |  |
|  |  | |  |  |  |
|  | | | |  |  |
|  | | | | | |
