# Supplementary material for: Intraoperative milrinone versus dobutamine in cardiac surgery patients: a retrospective cohort study on mortality
Source: Crit Care. 2018 Feb 26;22:51. doi: 10.1186/s13054-018-1969-1 (PMC5828330; doi:10.1186/s13054-018-1969-1)
Supplement: Supplementary file 2 — Table S2. presenting hemodynamic variables according to inotrope therapy pre and post CPB (DOCX 20 kb) [file 13054_2018_1969_MOESM2_ESM.docx]

|  | **Pre-CPB** | | | **Post-CPB** | | | | | **1. Post-operative hour** | | | | |
| --- | --- | --- | --- | --- | --- | --- | --- | --- | --- | --- | --- | --- | --- |
|  | CI  (IQR) | S_v_O_2_ (IQR) | MAP  (IQR) | CI  (IQR) | CI<2  % | S_v_O_2_  (IQR) | MAP  (IQR) | Lowest MAP <50mmHg  % | CI  (IQR) | CI<2  % | S_v_O_2_  (IQR) | MAP  (IQR) | Lowest MAP  <50 mmHg  % |
| **Dobutamine** | 2.0 (0.7) | 70.2  (11.6) | 70.3  (11.6) | 2.2  (0.8) | 23.2 | 69.5  (11.4) | 71  (9.9) | 32.7 | 2.2  (0.9) | 22.6 | 65.5  (12.4) | 74.0  (11.9) | 11.9 |
| **Milrinone** | 2.0  (0.6) | 67.3  (10.3) | 71.2  (11.2) | 2.2  (0.7) | 17.8 | 68.0  (11.4) | 67.9  (9.5) | 43.1 | 2.2  (0.6) | 24.3 | 65  (11.0) | 72.0  (11.9) | 13.8 |
| ***P* =** | 0.20 | 0.01 | 0.74 | 0.08 | 0.07 | 0.32 | < 0.01 | < 0.01 | 0.44 | 0.60 | 0.03 | 0.02 | 0.46 |

**Additional Table S2: Hemodynamic variables according to inotrope therapy**

Data are given as median (IQR) or %. Treatment groups were compared using chi-square tests for categorical parameters and Wilcoxon Mann-Whitney U test for non-parametric continuous parameters.

*Abbreviations: CPB* Cardiopulmonary bypass, *CI* Cardiac index (L/min/m^2^), *S_v_O_2_* Mixed venous oxygen saturation, *MAP* Mean arterial Pressure, *CI<2=* Frequency of patients where lowest CI measured within first hour after CPB or within 1.postoperative hour was below 2 L/min/m^2^ , *Lowest MAP* =frequency of patients where lowest MAP measured within first hour after CPB or within 1.postoperative hour was below 50 mmHg
